# First record of the genus *Echthronomas* Forster, 1869 (Hymenoptera, Ichneumonidae, Campopleginae) for the fauna of Ukraine

**DOI:** 10.3897/BDJ.2.e1006

**Published:** 2014-01-14

**Authors:** Alexander Varga

**Affiliations:** † I.I. Schmalhausen Institute of Zoology of National Academy of Sciences of Ukraine, Kiev, Ukraine

**Keywords:** Parasitoids, Ichneumonidae, Campopleginae, Ukraine, Ukrainian Carpathians, new records

## Abstract

The genus *Echthronomas* Forster, 1869 and two species, *Echthronomas facialis* (Thomson, 1887) and *Echthronomas quadrinotata* (Thomson, 1887), are recorded for the fauna of Ukraine for the first time. Descriptions and photographs of species are provided.

## Introduction

*Echthronomas* is a small genus of the subfamily Campopleginae (Hymenoptera: Ichneumonidae), represented by twelve species in the world, including four in the Western Palaearctic region (Yu et al. 2012). With the exception of *Echthronomas quadrinotata* (Thomson, 1887), *Echthronomas* species, which are endoparasitoids of lepidopteran larvae, are rarely collected (Horstmann 1987).

## Materials and methods

Specimens were collected by sweep netting on *Corylus avellana* in beech forest (Transcarpathia) and near branches of dead *Picea abies* in the mixed forest (Precarpathia). Specimens were identified using Horstmann's (Horstmann 1987) keys. Specimens were sent to Klaus Horstmann and he confirmed identification. Terminology followed Townes (1969). The generic diagnosis is that of Townes (1969) with minor modifications.

## Taxon treatments

### Echthronomas

Forster, 1869

Echthronomas Casinaria ochrostoma (Holmgren, 1860)

#### Diagnosis

Fore wing 5.3 to 8.0 mm long. Body short and stout. Eye margin indented opposite antennal socket. Clypeus small, its apex convex. Mandible short. Lower tooth of mandible a little shorter than upper tooth. Temple short. Mesopleurum matt and with strong punctures. Propodeum short, with or without well defined carinae, its basal transverse carina more or less distinct, other carinae not always present. Areola and petiolar areas, when defined, broadly confluent. Propodeal spiracle circular. Tarsal claws pectinate. Areolet pointed or petiolate. First metasomal segment slender. Glymmae present. Metasoma compressed. Thyridium subcircular, separated from base of the second tergite by 0.2 to 0.6 its diameter. Ovipositor as long as apical depth of metasoma. This genus is distinguished from other Campoplegine genera by combination of the following characters (Fig. 1).

### Echthronomas facialis

(Thomson, 1887)

#### Materials

- **Type status:** Other material. **Occurrence:** recordedBy: Varga A.; sex: 1 female; **Location:** country: Ukraine; stateProvince: Ivano-Frankivsk region, Bogorodchany district, Mochary; verbatimLocality: 5 km NE of Bogorodchany; verbatimElevation: 300–350 m; verbatimLatitude: 48° 50' N; verbatimLongitude: 24° 35' E; **Event:** eventDate: 19 July 2011
- **Type status:** Other material. **Occurrence:** recordedBy: Varga A.; sex: female; **Location:** country: Ukraine; stateProvince: Ivano-Frankivsk region, Bogorodchany district, Mochary; verbatimLocality: 5 km NE to Bogorodchany; verbatimElevation: 300-350 m; verbatimLatitude: 48° 50' N; verbatimLongitude: 24° 35' E; **Event:** eventDate: 10 June 2012

#### Diagnosis

*Female*. This species is easily distinguishable from all *Echthronomas* species in having a punctate propodeum, without traces of carinae, yellow frons (inner margins of eyes), face, clypeus, mandibles, malar space (Fig. 2a), tegula, scape and pedicel, black metasoma with tergites II–III apically yellowish and black hind femora (Fig. 2b). The other three species of *Echthronomas* have propodeum with traces of transverse carinae, entirely black frons, at least partly black face, at least partly red metasoma and red hind femora.

#### Biology

##### Hosts

Unknown.

### Echthronomas quadrinotata

(Thomson, 1887)

#### Materials

- **Type status:** Other material. **Occurrence:** recordedBy: Varga A.; sex: female; **Location:** country: Ukraine; stateProvince: Transcarpathian region, Rakhiv district, Kvasy; verbatimElevation: 630-650 m; verbatimLatitude: 48° 08' N; verbatimLongitude: 24° 16' E; **Event:** eventDate: 16 August 2009

#### Diagnosis

*Female*. This species is easily distinguishable from all Echthronomas species in having a transversely wrinkled propodeum, black clypeus with two yellow lateral spots, black face and malar space (Fig. 3a), black fore coxae with red (yellowish) spots, black metasoma with red postpetiole apically and tergites II–IV (Fig. 3b).

*Echthronomas tricincta* (Gravenhorst, 1829) differs from *Echthronomas quadrinotata* (Thomson, 1887) in having entirely yellow fore and mid coxae, clypeus, and malar space, partly yellow face, and a black metasoma with only red apically tergites II–IV. Another species, *Echthronomas ochrostoma* (Holmgren, 1860), is similar to *Echthronomas quadrinotata* (Thomson, 1887), but differs in having a yellow malar space and clypeus (sometimes with a small black central spot) (Horstmann 1987).

#### Biology

##### Hosts

*Eilema* sp. (Arctiidae) (Horstmann 1987).

## Supplementary Material

XML Treatment for Echthronomas

XML Treatment for Echthronomas facialis

XML Treatment for Echthronomas quadrinotata

## Figures and Tables

**Figure 1a.**
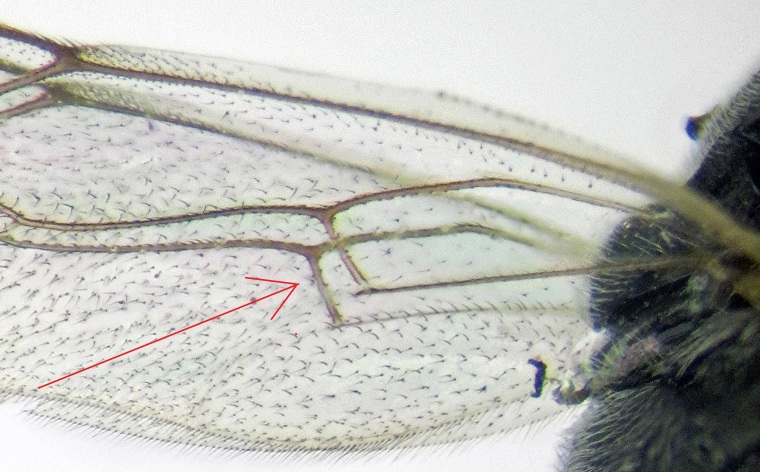
Nervellus.

**Figure 1b.**
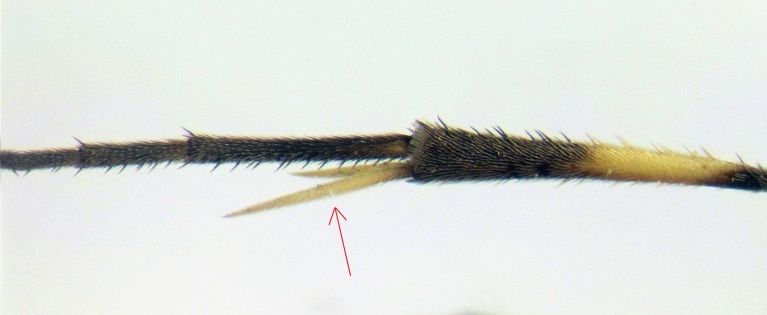
Spurs of hind tibia.

**Figure 2a.**
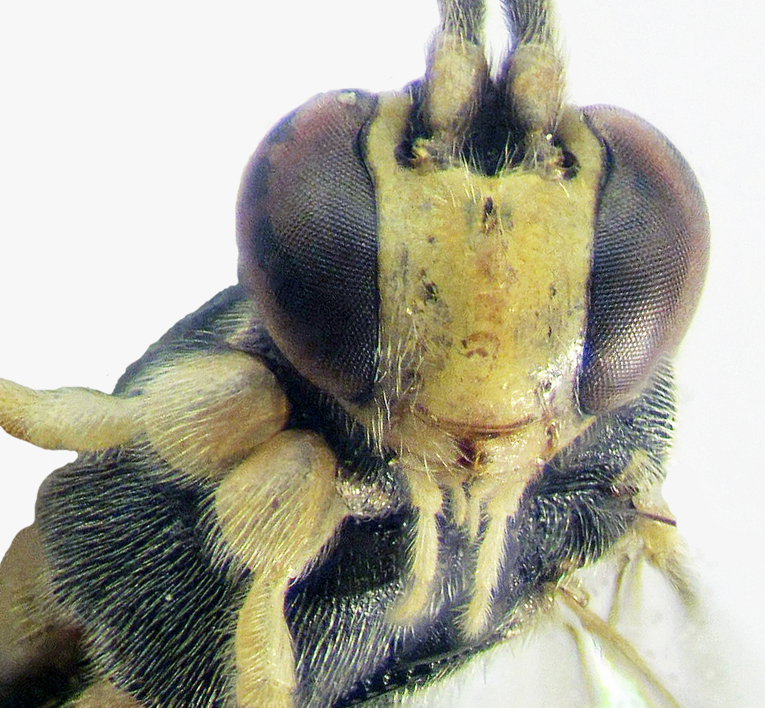
Head and fore coxae (frontal view).

**Figure 2b.**
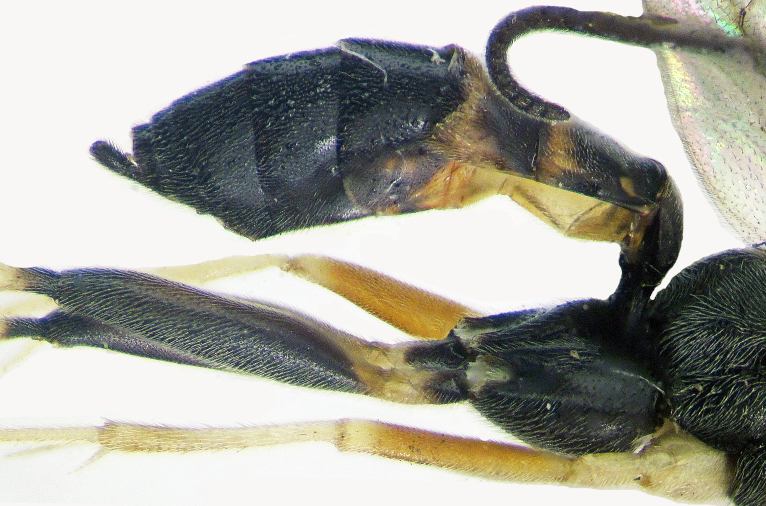
Metasoma and hind femora (lateral view).

**Figure 3a.**
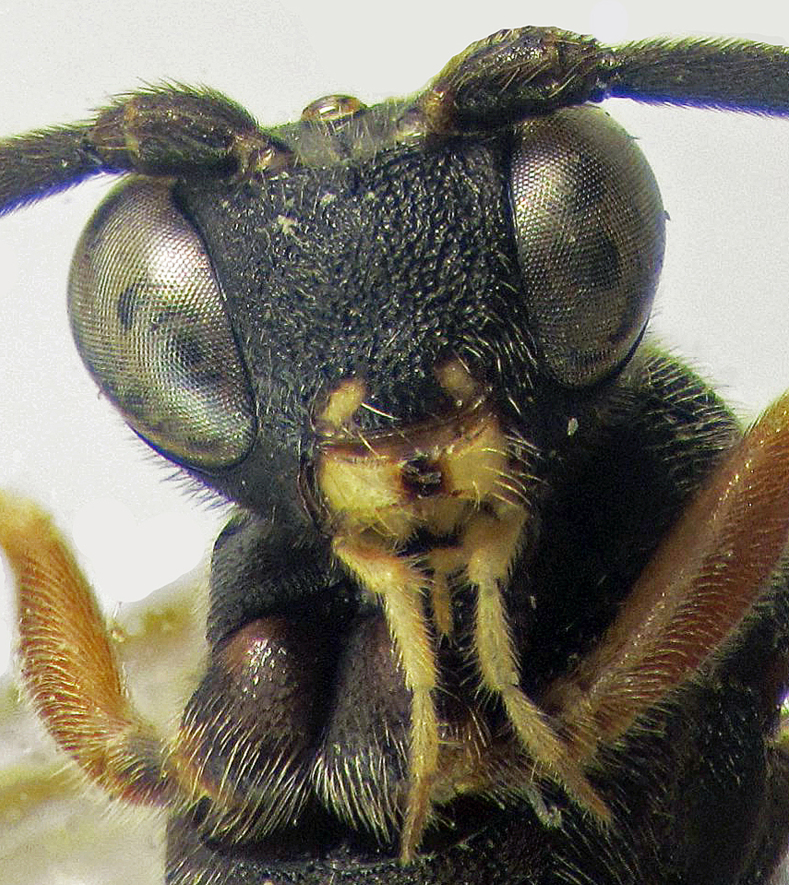
Head and fore coxae (frontal view).

**Figure 3b.**
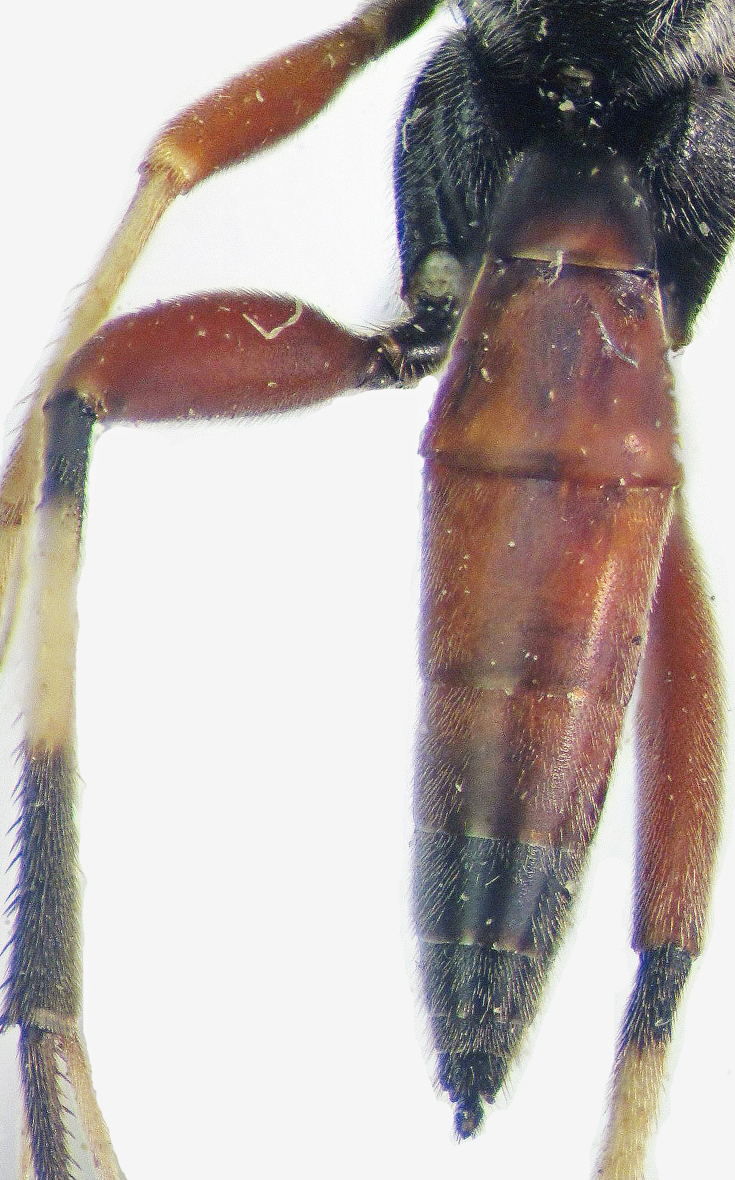
Metasoma and hind femora (dorsal view).
